# Supplementary material for: Angiogenesis is uncoupled from osteogenesis during calvarial bone regeneration
Source: Nat Commun. 2024 Jun 4;15:4575. doi: 10.1038/s41467-024-48579-5 (PMC11150404; doi:10.1038/s41467-024-48579-5)
Supplement: Supplementary file 1 — Supplementary Information [file 41467_2024_48579_MOESM1_ESM.pdf]

Angiogenesis is uncoupled from osteogenesis during calvarial bone regeneration

M. Gabriele Bixel[1#], Kishor K Sivaraj[1], Melanie Timmen[4], Vishal Mohanakrishnan[1], Anusha Aravamudhan[1], Susanne Adams[1], Bong Ihn Koh[1], Hyun-Woo Jeong[2], Kai Kruse[3], Richard Stange[4], and Ralf H. Adams[1#]

Affiliations:

[1]Max Planck Institute for Molecular Biomedicine, Department of Tissue Morphogenesis, and University of Münster, Faculty of Medicine, D-48149 Münster, Germany.
[2]Max Planck Institute for Molecular Biomedicine, Sequencing Core Facility, D-48149 Münster, Germany.
[3]Max Planck Institute for Molecular Biomedicine, Bioinformatics Service Unit, D-48149 Münster, Germany.
[4]Department of Regenerative Musculoskeletal Medicine, Institute of Musculoskeletal Medicine, University Hospital Münster, D-48149, Münster, Germany.

[#]Authors for correspondence

M. Gabriele Bixel
Department of Tissue Morphogenesis
Max-Planck-Institute for Molecular Biomedicine and University of Münster
D–48149 Münster, Germany
mgbixel@mpi-muenster.mpg.de
Phone: +49 251 70365 430; Fax: +49 251 70365 499

Ralf H. Adams
Department of Tissue Morphogenesis
Max-Planck-Institute for Molecular Biomedicine and University of Münster
D–48149 Münster, Germany
ralf.adams@mpi-muenster.mpg.de
Phone: +49 251 70365 410; Fax: +49 251 70365 499

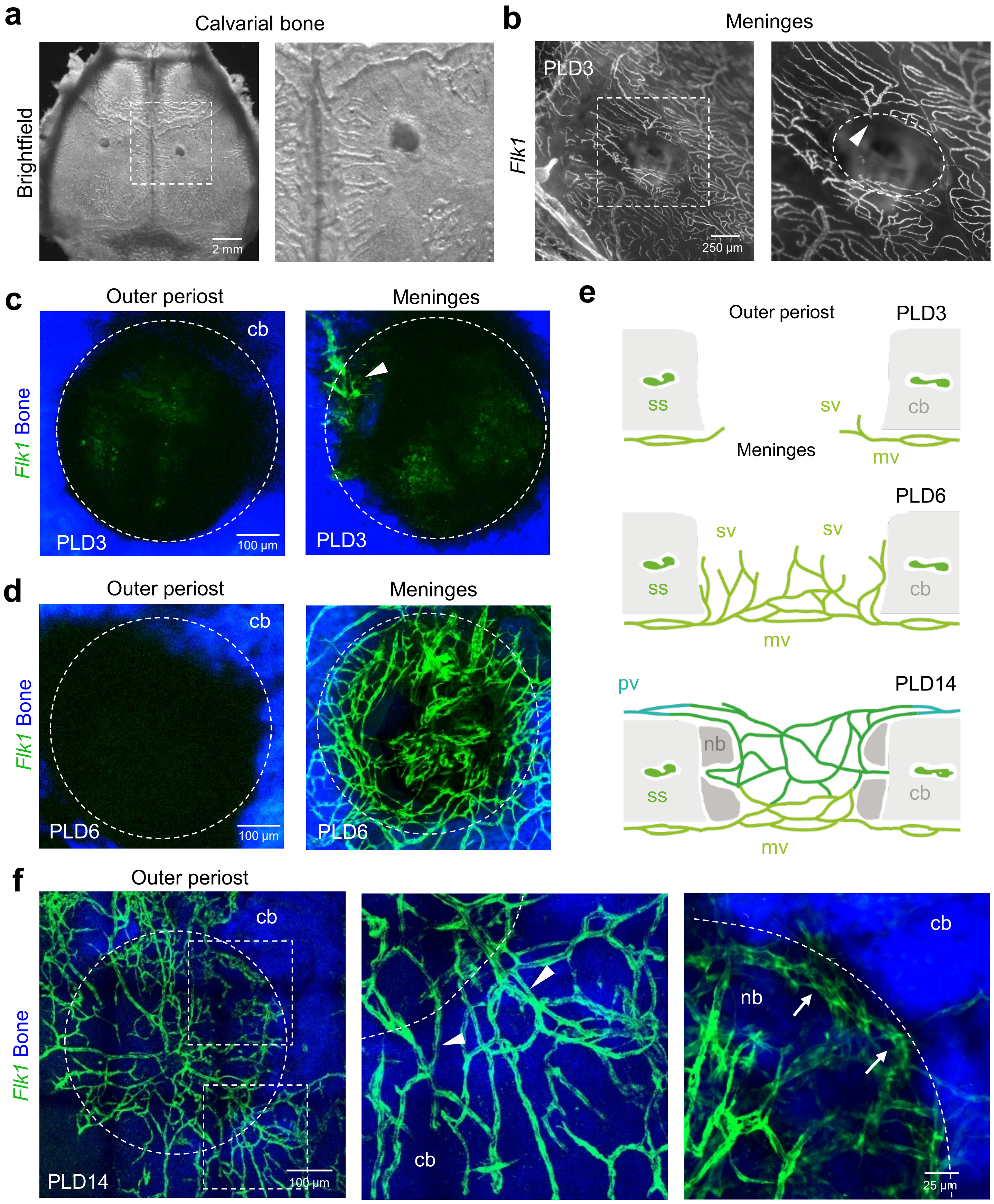

Supplementary Fig. 1

Supplementary Figure 1. Regenerating microvessels emerge from the meningeal vasculature after calvarial bone injury.

a. Stereomicroscopic bright field image of whole mount calvarial bone with lesion injuries in the parietal bones.
b. Stereomicroscopic fluorescence image showing meningeal vasculature of calvarial bone whole mount from *Flk1-GFP*$^{+}$ transgenic mice at PLD3. Note the absence of meningeal microvessels at the site of the calvarial bone lesion due to the drill hole injury.
c, d. Multiphoton microscopic image of the calvarial bone of *Flk1-GFP*$^{+}$ transgenic mice at PLD3 (**c**) and PLD6 (**d**). Outer perivascular side (left) and inner meningeal side (right) are shown. Arrow head points to an early *Flk1-GFP*$^{+}$ (green) sprout from the meningeal vasculature into the lesion. The injured SHG^{+} calvarial bone is shown in blue. The calvarial bone lesion appears as dark area.
e. Schematic cross section showing early stages of calvarial bone healing with sprouting vessels (sv) originating from the meningeal vasculature and form an early vascular plexus. Sprouts continue to extend from the vascular plexus and connect with periosteal vessels at the outer edge of the bone.
f. Intravital multiphoton image showing numerous vascular connections between the newly formed *Flk1-GFP*$^{+}$ microvasculature and periosteal microvessels at PLD14. The injured calvarial bone tissue (cb) and newly formed bone (nb) are visualized by SHG imaging. Overview image (left) and zoom-in images with periosteal vessel connections (middle, arrowheads) and microvessels aligned with the injured calvarial bone in the lesion (right, arrows).
Reproducibility was ensured by n=3 independent experiments.

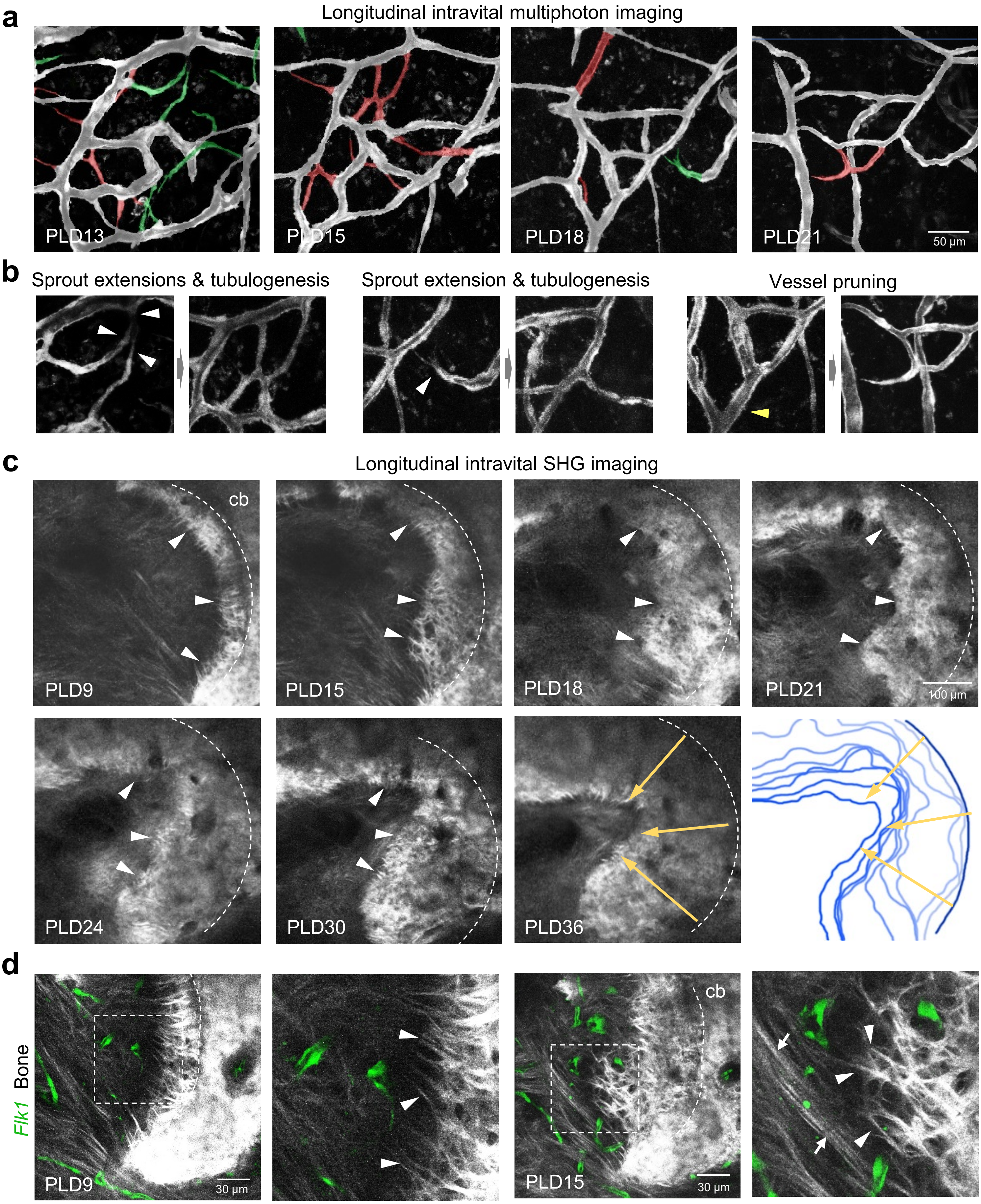

Supplementary Fig. 2

Supplementary Figure 2. Longitudinal intravital multiphoton imaging of vascular remodeling and calvarial bone regeneration.

a. Longitudinal intravital multiphoton microscopy of a selected area showing sprouting, remodeling and pruning of regenerating *Flk1-GFP*$^{+}$ microvasculature after calvarial lesion injury over a 1-week period (PLD13-PLD21). Sprouting (green) and pruning (red) vessels were color-labeled.

b. Longitudinal imaging allows tracking of sprouting and pruning events in the remodeling vasculature. White arrowheads indicate vascular sprouts that form luminized vascular connection. Yellow arrowhead indicates a pruning vessel detaching from an exciting vessel.

c. Longitudinal intravital multiphoton micrograph of progressive calvarial bone repair after lesion injury using SHG imaging over a 4-week period (PLD9-PLD36). Arrowheads indicate growing SHG^{+} bone edge. Blue lines in the lower right image indicate the front of the growing bone at different stages of healing. Yellow arrows indicate bone growth at three selected sites. Note that the local rate of bone formation varies considerably.

d. Intravital multiphoton images and magnified views at PLD9 (left) and PLD15 (right) showing thin and parallel SHG^{+} collagen fibres (arrows) in the uncalcified lesion and thicker, crisscrossing SHG^{+} collagen fibres at the front and in the new bone matrix. GFP^{+} microvessels are frequently found in close proximity to the SHG^{+} bone matrix. A corresponding 3D representation of PLD15 is shown in Supplementary Movie 2. Reproducibility was ensured by n=3 independent experiments.

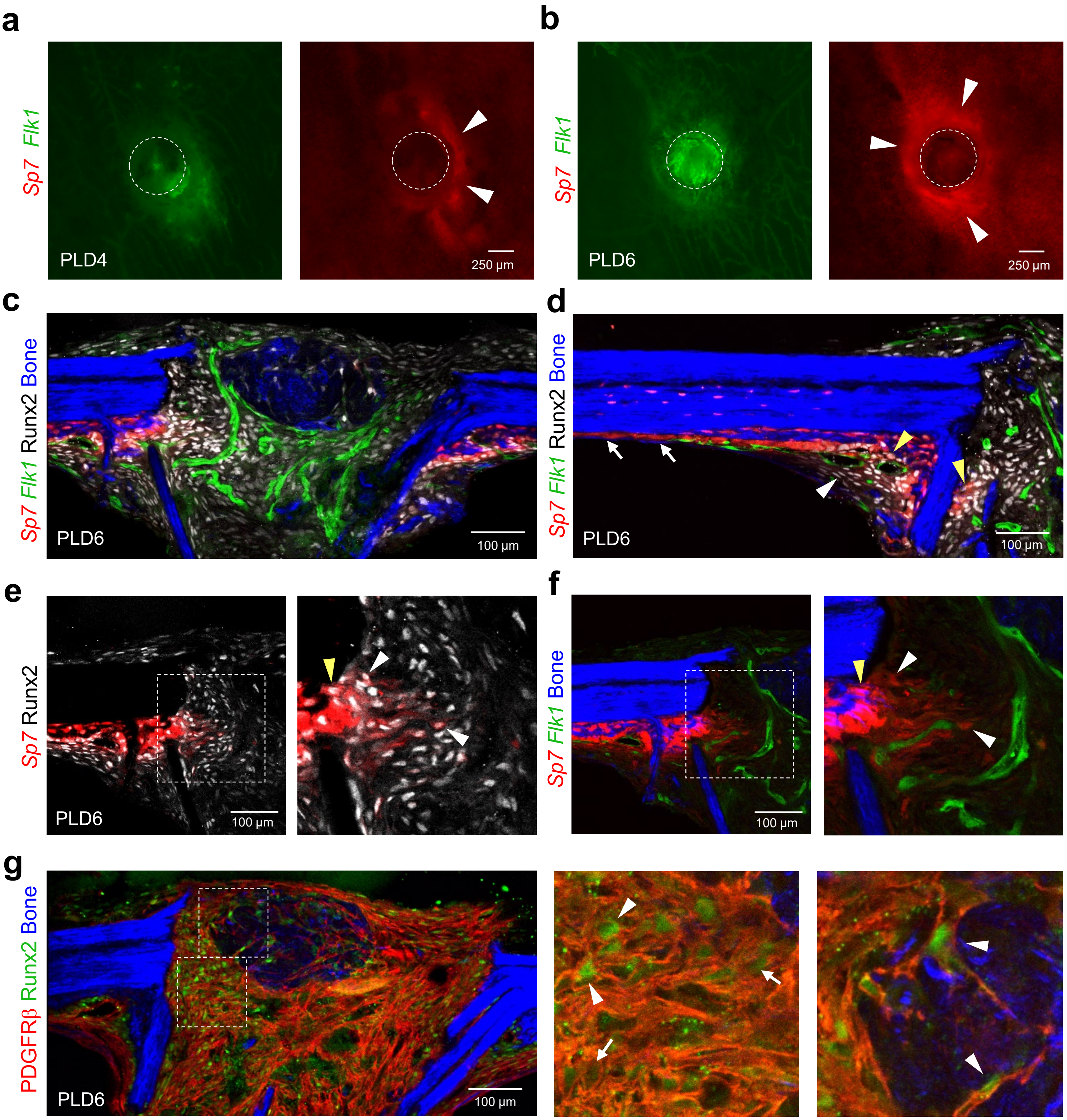

Supplementary Fig. 3

Supplementary Figure 3. Osteoprogenitors emerge from the periosteum and collectively invade the calvarial bone lesion.

a-b. Stereomicroscopic fluorescence image of calvarial bone wholemounts from *Flk1-GFP, Sp7-mCherry* double reporter mice at PLD4 (**a**) and PLD6 (**b**). Arrowheads indicate increased expression of *sp7-mCherry* around the lesion area at PLD4, which is even more pronounced at PLD6.

c, d. Multiphoton image of a cross section showing calvarial bone lesion at PLD6 from a central view (**c**) and distal view (**d**) of *Flk1-GFP, Sp7-mCherry* double reporter mice stained for Runx2 expression. Overview images in **c, d** (maximum intensity projections) show GFP^{+} (green) microvasculature, Runx2^{+} (white) progenitor cells, Osx^{+} osteoblasts and progenitors, and SHG^{+} calvarial bone tissue. White arrowhead indicates Runx2^{+}Osx$^{+\ low}$ osteoprogenitor and yellow arrowhead indicates Runx2^{+}Osx$^{+\ high}$ osteoprogenitor. White arrows point to Osx^{+} cells at a site distant from the lesion without expanded periost layer in response to lesion injury.

e, f. Multiphoton image (single plane of **c**) of a calvarial bone cross section at PLD6 with zoom-in (right). White arrowheads indicate Runx2^{+}Osx$^{+\ low}$ osteoprogenitor and yellow arrow head to Runx2^{+}Osx$^{+\ high}$ osteoprogenitor. Note that spindle-shaped Runx2^{+}Osx$^{+\ low}$ osteoprogenitors collectively invade the calvarial bone lesion, often in proximity to GFP^{+} microvessels.

g. Multiphoton image of a calvarial bone cross section at PLD6 with overview image (left) and zoom-in images (right) showing Runx2^{+} (green) and PDGFRβ^{+} (red) cells in the calvarial lesion. SHG^{+} calvarial bone is shown in blue. Note that PDGFRβ^{+} stromal cells are highly abundant in the lesion and often express both, PDGFRβ and Runx2. Arrowheads point to PDGFRβ^{+}Runx2^{+} cells with spindle cell morphology, while arrows point to PDGFRβ^{+}Runx2^{-} cells.

Reproducibility was ensured by n=3 independent experiments.

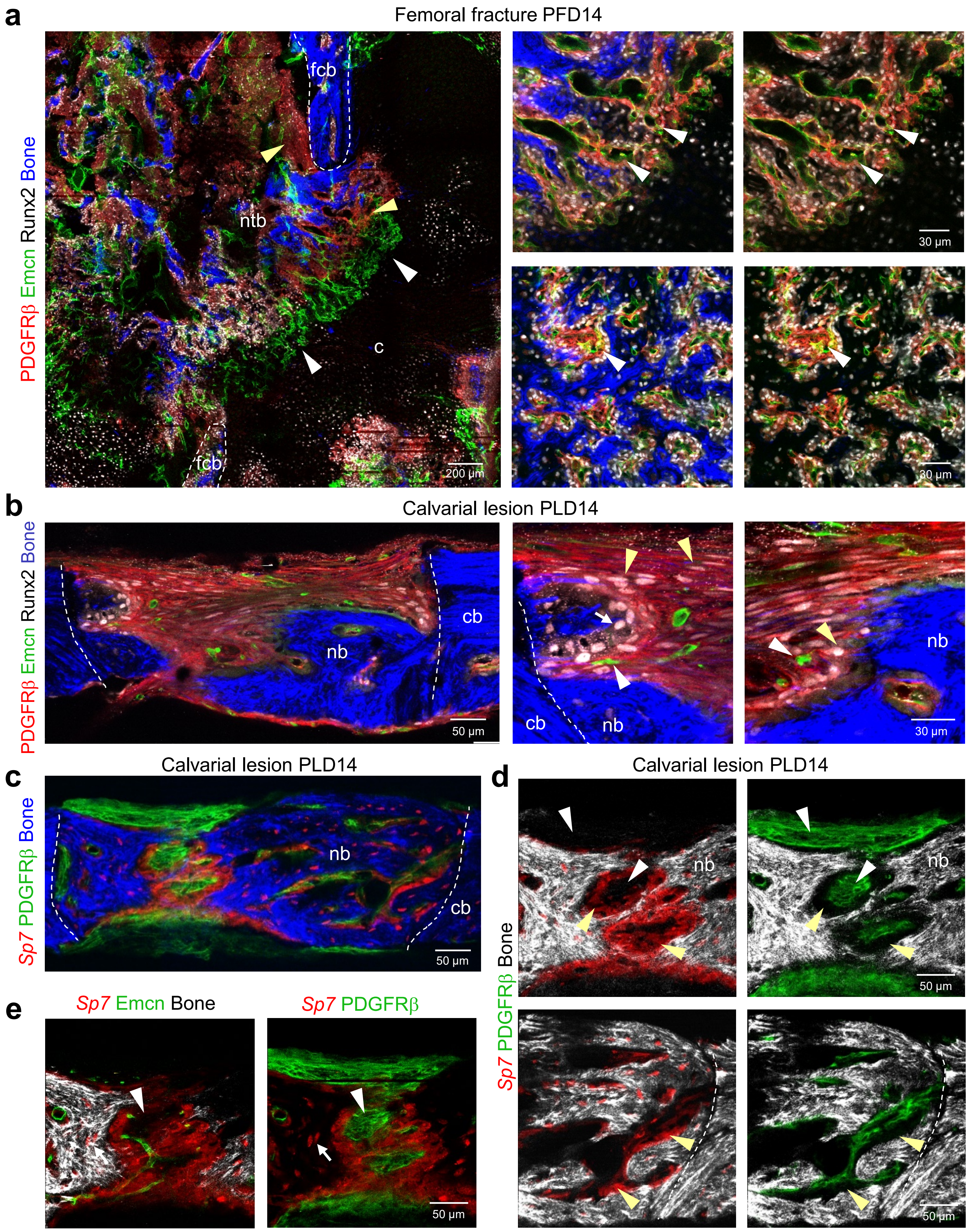

Supplementary Fig. 4

Supplementary Figure 4. PDGFRβ$^+$ stromal cells are highly abundant in calvarial bone lesions at PLD14.

a. Tile-scan multiphoton microscopy of PFD14 femoral fracture. Overview image shows maximum intensity projections of Emcn$^+$ (green) microvasculature, Runx2$^+$ (white) cells, PDGFRβ$^+$ (red) cells and SHG$^+$ (blue) newly formed trabecular-like woven bone (ntb) and fractured compact bone (fcb). Zoom-in views show PDGFRβ$^+$ cells associated with Emcn$^+$ microvessels at the chondro-osseous junction (top) and in the calcified callus with early SHG$^+$ bone matrix deposition (bottom). Note that PDGFRβ$^+$ stromal cells are associated with Emcn$^+$ microvessels and frequently express both, PDGFRβ and Runx2.
b. Multiphoton image of a calvarial bone cross section at PLD14 with overview (left) and zoom-in images (right) showing Runx2$^+$ (white) and PDGFRβ$^+$ (red) cells in the calvarial lesion. SHG$^+$ calvarial bone is shown in blue. Note that PDGFRβ$^+$ stromal cells are highly abundant in the vascularized lesion and frequently express both, PDGFRβ and Runx2 with elongated nuclei (yellow error heads). Runx2$^+$ cells with round nuclei (white arrow) are close to the growing bone edge.
c-e. Multiphoton microscopy showing PDGFRβ$^+$ stromal cells and Sp7$^+$ osteoblasst and progenitor cells in the calvarial bone lesion of PLD14 with overview image (**c**) and zoom-in images (**d-e**). SHG$^+$ calvarial bone is shown in blue and white. Note that PDGFRβ$^+$ cells are negative for Sp7 (white arrowhead) and occupy complementary areas to Sp7$^+$ osteoblasts. Sp7$^+$ osteoblasts deposit SHG$^+$ bone matrix and transform into bone-embedded Sp7$^+$ osteocytes (white arrow).
Reproducibility was ensured by n=3 independent experiments.

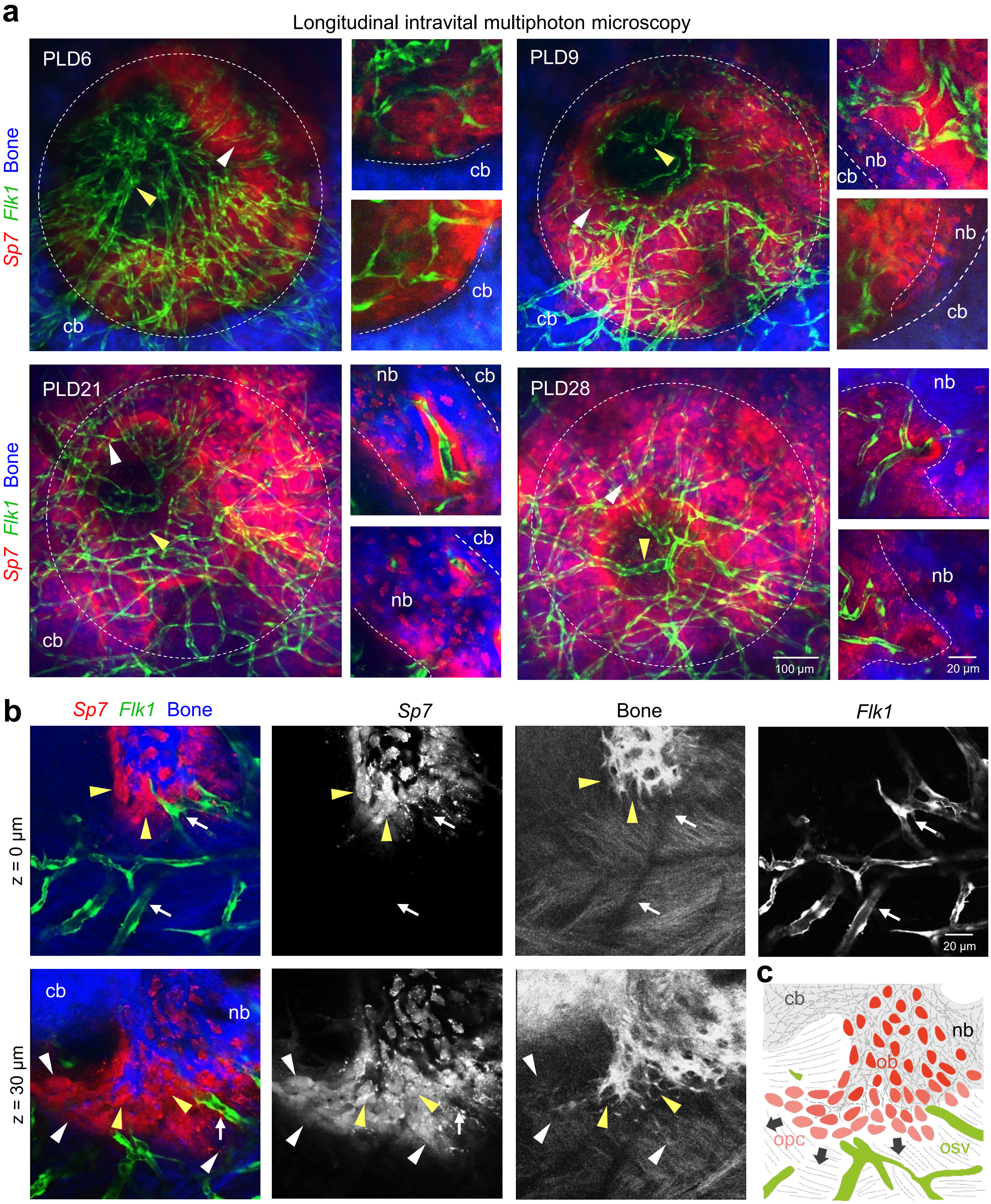

Supplementary Fig. 5

Supplementary Figure 5. Bone-forming osteoblasts and osteoprogenitors collectively invade the vascularized lesion during calvarial bone regeneration.

a. Longitudinal intravital multiphoton microscopy showing the gradual progression of calvarial bone repair in *Flk1-GFP sp7-mCherry* double transgenic mice after lesion injury. Overview images show maximum intensity projections of *Sp7-mCherry*$^{+}$ (red) osteoblasts and progenitor cells that densely lining the growing SHG^{+} (blue) calvarial bone in proximity to sprouting and remodeling *Flk1*$^{+}$ (green) microvessels. Note that early *Flk1-GFP*$^{+}$ vessels have invaded and vascularized the entire lesion, while early *Sp7-mCherry* $^{+}$ osteoblasts and progenitor cells line the growing SHG^{+} bone edge as a multicellular layer. Enlarged views on the right show i) early *Sp7-mCherry* $^{+}$ osteoblastic cells lining the injured SHG^{+} calvarial bone (cb) close to GFP^{+} microvessels at PLD6, ii) newly formed SHG^{+} calvarial bone (nb) with early enclosed *Sp7-mCherry*$^{+}$ osteocytes, *Sp7-mCherry*$^{+}$ osteoblastic cells and *Flk1-GFP*$^{+}$ microvessels close to the growing bone edge at PLD9, iii) expanded SHG^{+} calvarial bone with enclosed *Sp7-mCherry*$^{+}$ osteocytes, early BM cavities and enclosed *Flk1-GFP*$^{+}$ microvessels at PLD21, and iv) expanded SHG^{+} calvarial bone edge at PLD28. White arrows point to vessels close to *Sp7-mCherry*$^{+}$ osteoblastic cells at the growing bone edge. Yellow arrows point to microvessels in the uncalcified lesion area not associated with osteoprogenitors.

b. Intravital multiphoton micrograph showing a zoom-in view of the growing SHG^{+} calvarial bone edge at PLD14. Two different image planes are shown (z = 0 µm, z = 30 µm). Early *Sp7-mCherry*$^{+}$ osteoblasts and progenitors form a multicellular sheet at the front of the growing bone where they collectively invade the vascularized lesion site. White arrowheads indicate early invading *Sp7-mCherry*$^{+}$ osteoprogenitors that are not surrounded by SHG^{+} fibres. Yellow arrowheads point to *Sp7-mCherry*$^{+}$ osteoblastic cells surrounded by first SHG^{+} fibres of the new bone matrix. Note that their cell bodies are excluded and appear as dark spots. White arrows point to *Flk1*$^{+}$ microvessels. Dark arrows indicate the direction of the growing bone.

c. Schematic of a growing calvarial bone (nb) lined by a multicellular layer of osteoprogenitor cells (opc), osteoblasts (ob) growing into a vascularized lesion with adjacent osteogenic and remodeling microvessels (osv). cb: calvarial bone.

Reproducibility was ensured by n=3 independent experiments.

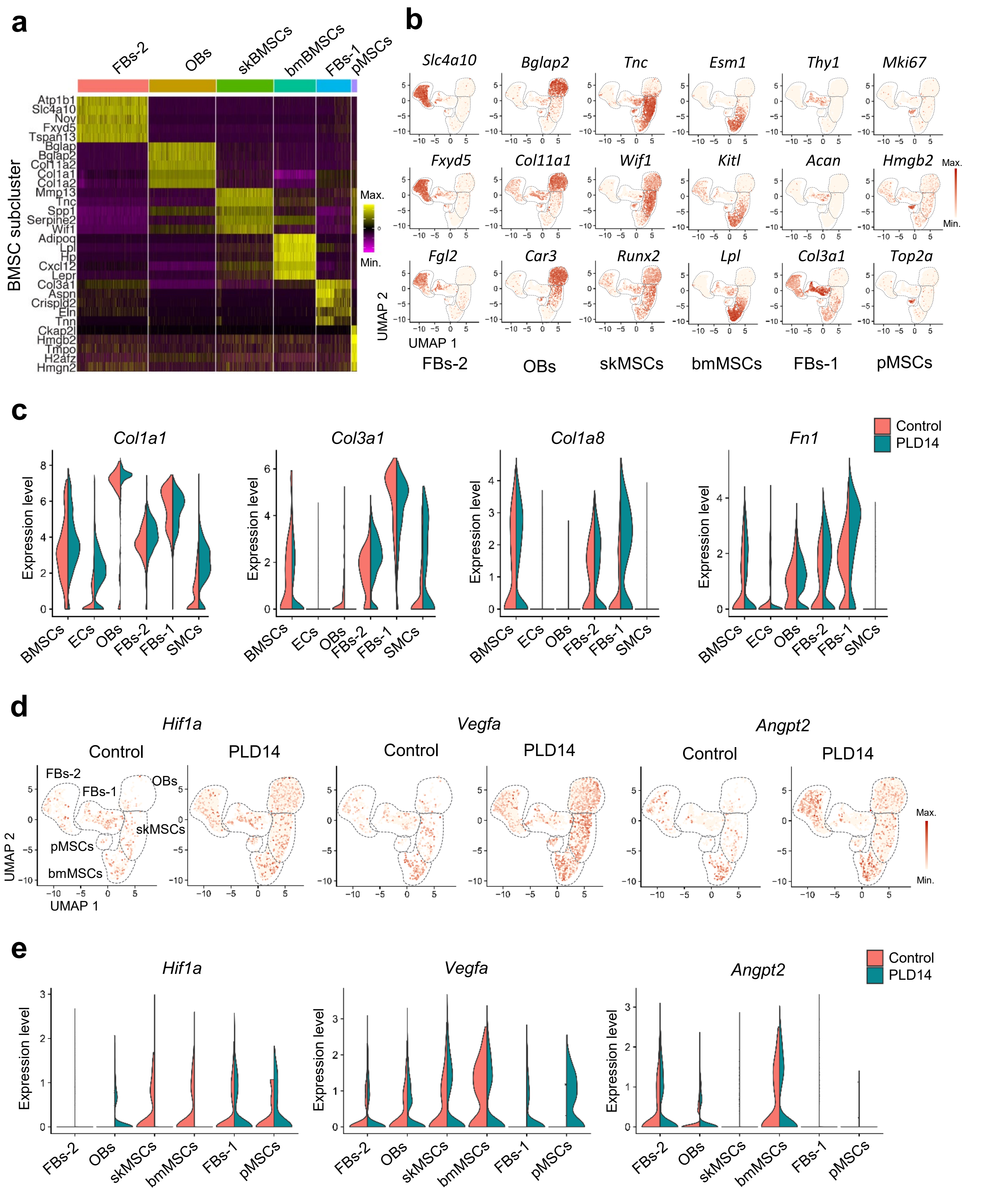

Supplementary Fig. 6

Supplementary Figure 6. Single-cell RNA-sequencing analysis of BMSC subcluster from calvarial bone lesions. Related to Figure 8

a. Heat map showing the top cell marker genes of the cell populations of the BMSC subcluster shown in **Figure 8e**.
b. UMAP plots showing three cell-specific marker genes for each cell population shown in **a**.
c. Violin plots showing the expression of *Col1a1, Col3a1, Col8a1* and *Fn1* in the BMSC subcluster.
d. BMSC subcluster shown in **Figure 8e** data showing expression of *Hif1a*, *Vegfa* and *Angpt2* in UMAP plots of control and PLD14.
e. Violin plots showing *Hif1a*, *Vegfa* and *Angpt2* gene expression in BMSC subcluster.

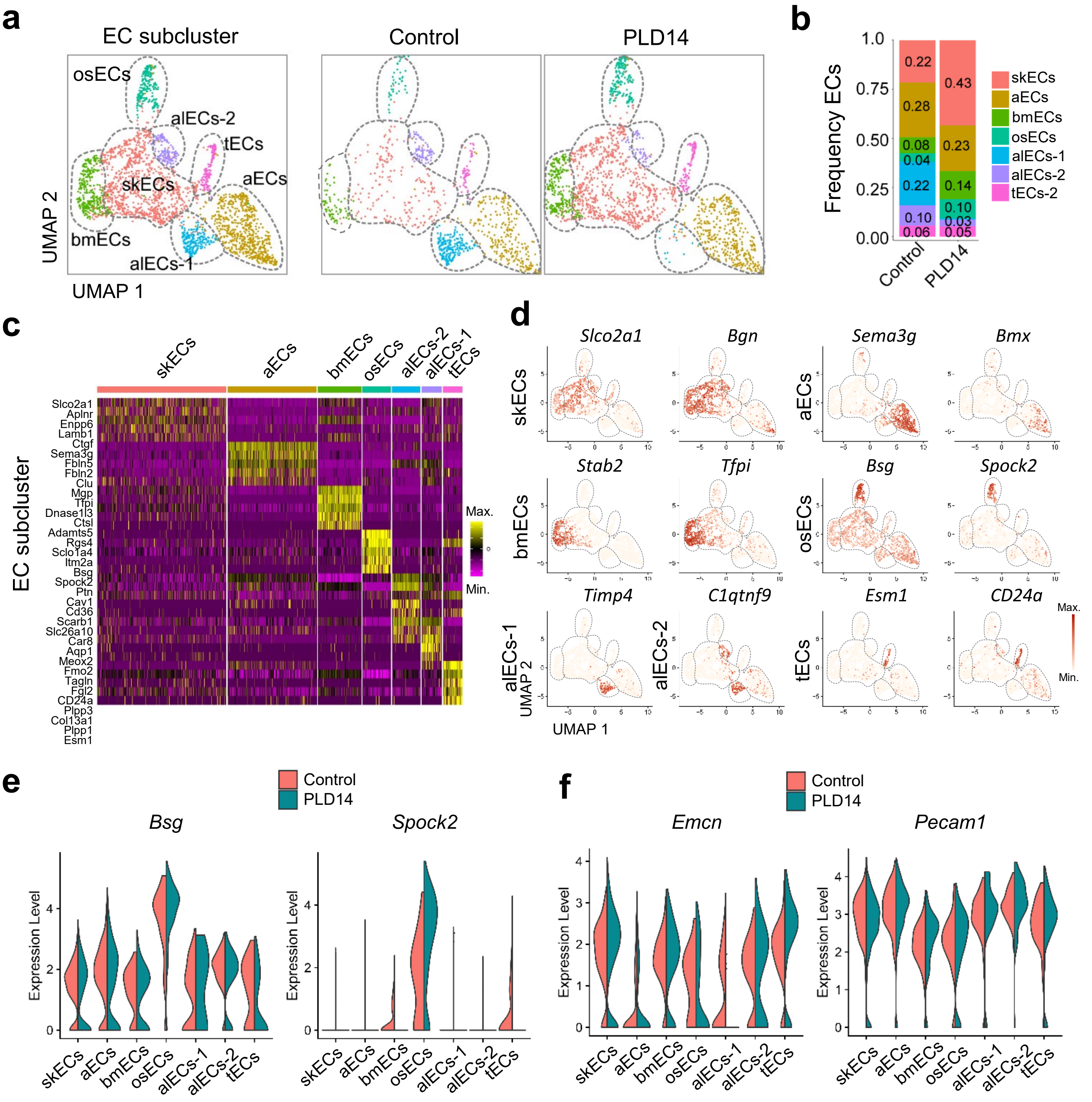

Supplementary Fig. 7

Supplementary Figure 7. Single-cell RNA-sequencing analysis of EC subcluster from calvarial bone lesions.

a. UMAP plots showing color-coded merged EC subcluster from calvarial bones at PLD14 (left), group-selected color-coded ECs subcluster from controls (middle) and calvarial bone lesions (right).

b. Frequency plots of color-coded EC subclusters at PLD14.

c. Heat map showing the top cell marker genes of each cell population.

d. UMAP plots showing two cell-specific marker genes for each cell population.

e. Violin plots showing *Bsg* and *Spock2* gene expression in the EC subcluster.

f. Violin plots showing *Emcn* and *Pecam1* gene expression in the EC subcluster.

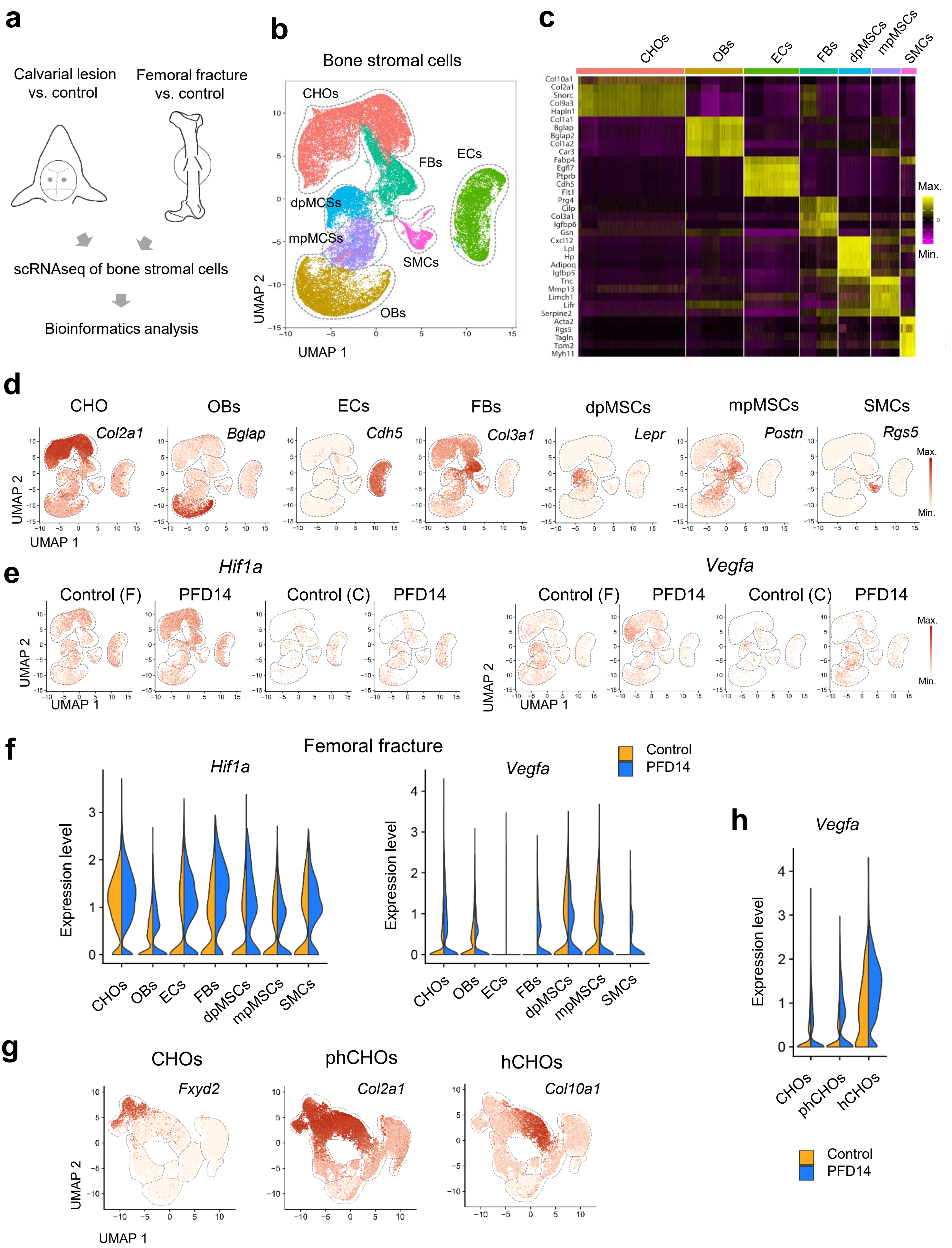

Supplementary Fig. 8

Supplementary Figure 8. Single-cell RNA-sequencing analysis of bone non-hematopoietic cells from calvarial lesion and femoral fractures.

a. Preparation of calvarial and femoral bones at PLD14 and PFD14 for scRNA-seq data analysis.

b. UMAP plots showing color-coded merged BMSC subcluster from injured calvarial and femoral bones at PLD14 and PFD14, respectively.

c. Heat map showing the top cell marker genes of each cell population.

d. UMAP plots showing cell-specific marker genes for each cell population.

e. BMSC subcluster showing expression of *Hif1a* (left) *and Vegfa* (right) in UMAP plots from calvarial lesions at PLD14, femoral fractures at PFD14 and their corresponding controls.

f. Violin plots showing *Hif1a* and *Vegfa* gene expression in the EC subcluster.

g. UMAP plots showing chondrocyte-specific marker genes in the EC subcluster.

h. Violin plots showing *Vegfa* gene expression in different CHO subcluster.

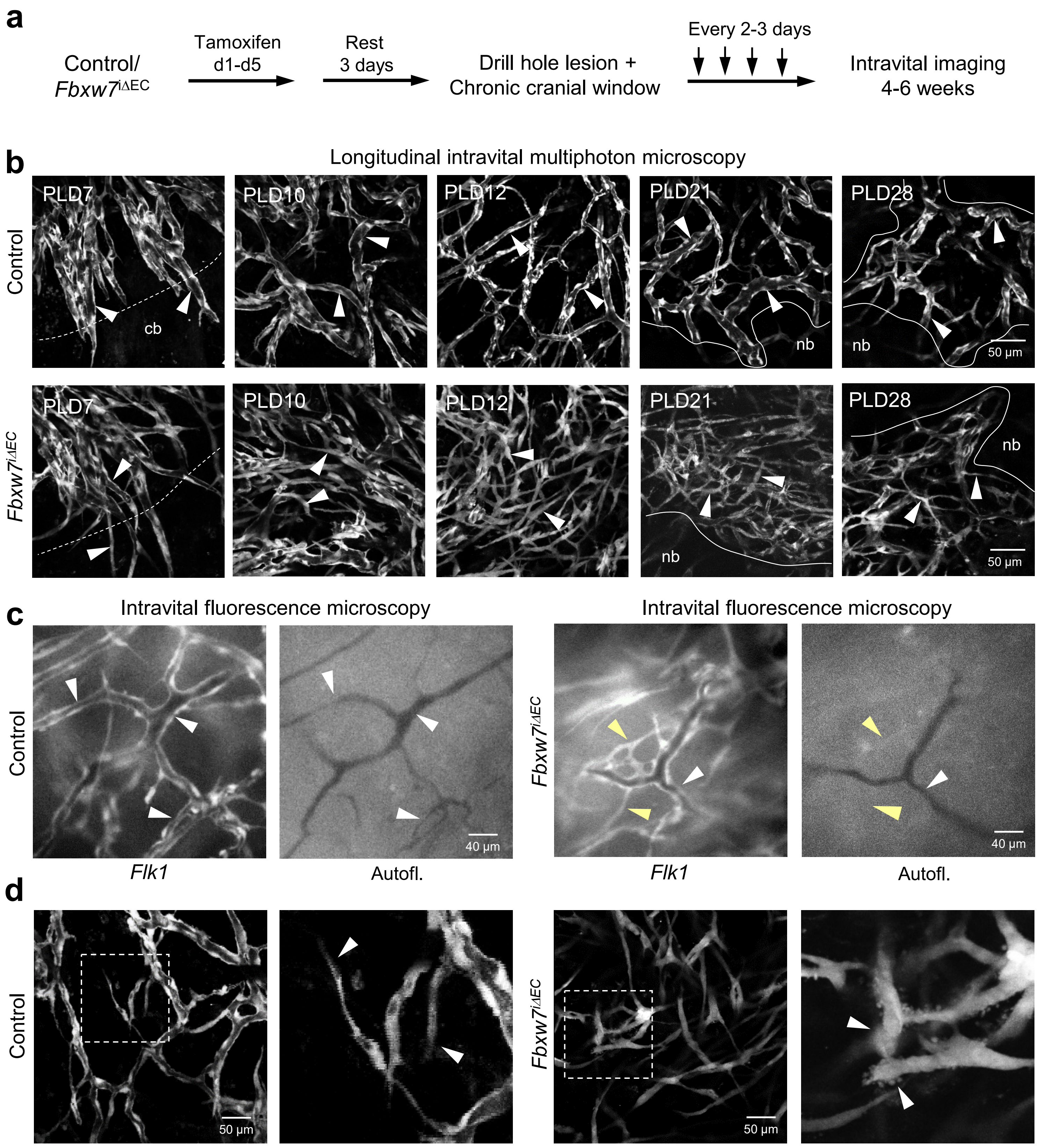

Supplementary Fig. 9

Supplementary Figure 9. Notch activation promotes sprouting and non-perfused microvessels formation after calvarial bone injury.

a. Experimental procedure showing tamoxifen-inducible *Fbxw7* deletion in ECs with Cdh5CreERT2 transgenic mice in *Flk1-GFP* transgenic background, followed by calvarial drill hole and cranial window surgery for repeated intravital multiphoton imaging.
b. Longitudinal intravital multiphoton micrograph showing sprouting and remodeling of *Flk1-GFP*$^{+}$ microvasculature in control and *Fbxw7* $^{i\Delta EC}$ *Flk1-GFP* transgenic mice from PLD7 to PLD28. Dotted lines at PLD7 indicate injured calvarial bone edge (cb). Straight lines at PLD21 and PLD28 indicate the growing bone edge (nb). Arrowheads indicate lumen-containing (control) and non-lumen-containing (mutant) *Flk1-GFP*$^{+}$ microvessels.
c. Intravital fluorescence micrographs of control (left) and *Fbxw7* $^{i\Delta EC}$ (right) *Flk1-GFP* transgenic mice showing GFP^{+} microvessels (right) and perfused (control) and non-perfused (mutant) microvessels visualized by autofluorescent red blood cells at PLD14. White arrowheads indicate perfused microvessels, yellow arrowheads indicate non-perfused microvessels.
d. Intravital multiphoton microscopy showing tip cell morphology of vascular sprouts from control (left) and *Fbxw7* $^{i\Delta EC}$ (right) mice at PLD12. Arrow heads point individual tip cells. Reproducibility was ensured by n=3 independent experiments.

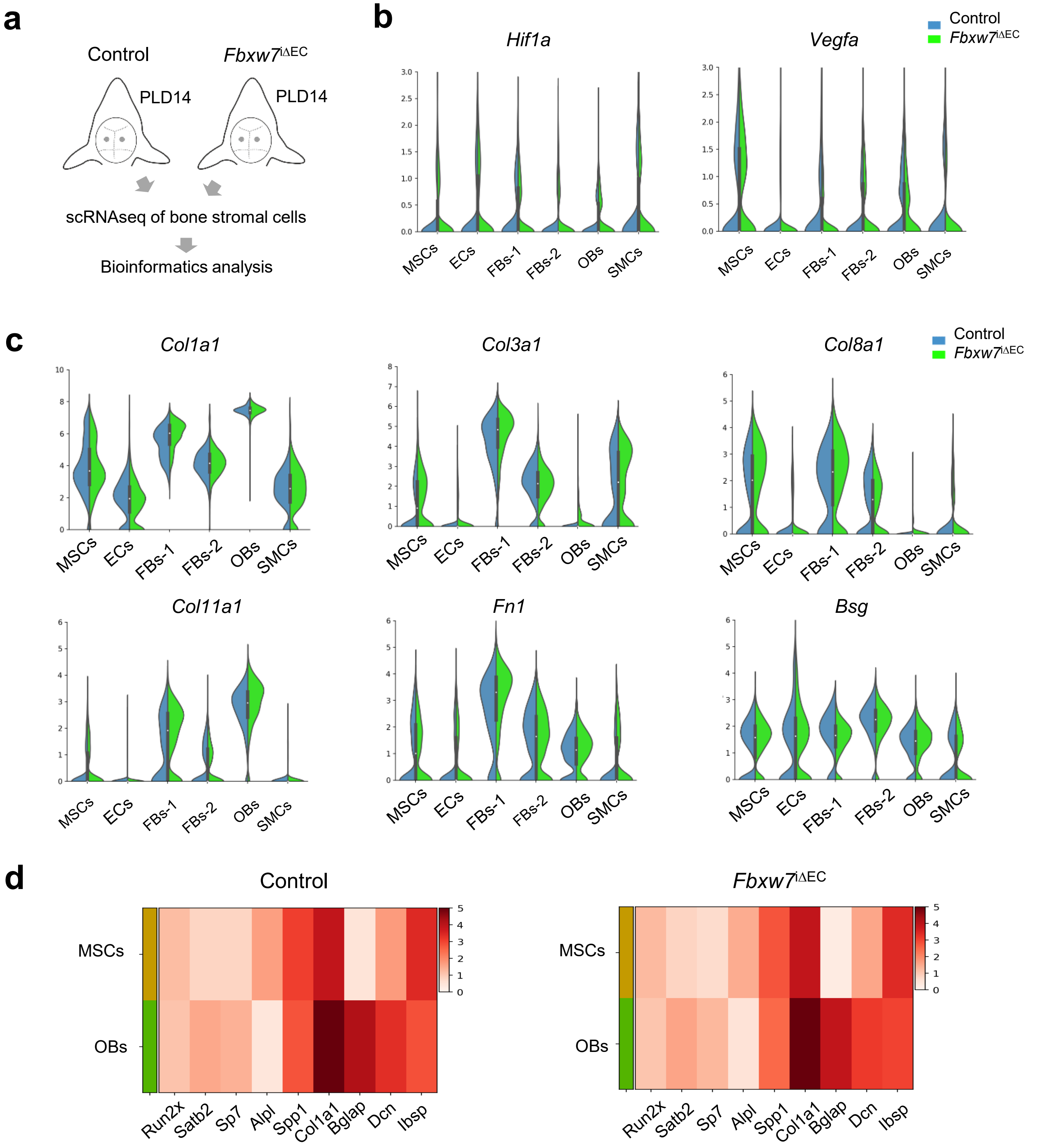

Supplementary Fig. 10

Supplementary Figure 10. Single-cell RNA-sequencing analysis of bone non-hematopoietic cells from control and *Fbxw7* $^{i\Delta EC}$ after calvarial lesion injury.

a. Preparation of control and *Fbxw7* $^{i\Delta EC}$ mutant calvarial bones at PLD14 for scRNA-seq data analysis.

b. Violin plots showing *Hif1a* and *Vegfa* gene expression in non-hematopoietic cells from control and *Fbxw7* $^{i\Delta EC}$ mutant calvarial bones at PLD14.

c. Violin plots showing *Col1a1, Col3a1, Col8a1, Col11a1, Fn1* and *Bsg* gene expression in non-hematopoietic cells from control and *Fbxw7* $^{i\Delta EC}$ mutant calvarial bones at PLD14.

d. Heatmaps showing expression of MSC and OB differentiation markers in non-hematopoietic cells from control and *Fbxw7* $^{i\Delta EC}$ mutant calvarial bones at PLD14.
