# Supplementary material for: Angiogenesis is uncoupled from osteogenesis during calvarial bone regeneration
Source: Nat Commun. 2024 Jun 4;15:4575. doi: 10.1038/s41467-024-48579-5 (PMC11150404; doi:10.1038/s41467-024-48579-5)
Supplement: Supplementary file 3 — Description of Additional Supplementary Files [file 41467_2024_48579_MOESM3_ESM.pdf]

Description of Additional Supplementary Files

File Name: Supplementary Movie 1
Description: Calvarial bone regeneration with associated microvessels. Related to Figure 2a.
Intravital multiphoton microscopy showing newly formed calvarial bone matrix with closely associated Flk1-GFP+ microvessels at PLD14. 3D representation of the injured SHG+ (white) calvarial bone edge (upper left) and the newly formed SHG+ bone adjacent in the lesion with closely associated Flk1-GFP+ (green) microvessels. Dimensions: 400x400x100 µm.

File Name: Supplementary Movie 2
Description: Expanding bone matrix at the regenerating bone front. Related to Supplementary Figure 2d.
Intravital multiphoton microscopy showing the SHG+ crisscrossing fiber network of the newly expanding calvarial bone matrix with closely associated Flk1-GFP+ microvessels at PLD14. 3D stack with single planes showing the edge of the injured SHG+ (white) calvarial bone (top left) and adjacent newly formed SHG+ bone with closely associated Flk1-GFP+ (green) microvessels. Dimensions: 80x80x50 µm.

File Name: Supplementary Movie 3
Description: Blood flow dynamics in sprouting vessels of the early vascular plexus after calvarial bone injury. Related to Figure 3a.
Blood flow through sprouting Flk1-GFP+ (green) microvessels and the early Flk1-GFP+ vascular plexus at the outer edge of the calvarial lesion at PLD7 visualized by intravital multiphoton imaging after intravenous injection of TexasRed-dextran (red). Note that the non-perfused luminal spaces of the vascular sprouts contain numerous blood cells that are stationary; but passively moved by the nearby blood circulation. Dimensions: 60x50 µm.

File Name: Supplementary Movie 4
Description: Blood flow dynamics in the early vascular plexus after calvarial bone injury. Related to Figure 3a.
Blood flow through the early Flk1-GFP+ vascular plexus at the outer edge of the calvarial lesion at PLD7 visualized by intravital multiphoton imaging after intravenous injection of TexasRed-dextran (red). Note the highly heterogeneous blood flow dynamics in the different vessel segments. Dimensions: 60x50 µm.
