# Supplementary material for: Angiogenesis is uncoupled from osteogenesis during calvarial bone regeneration
Source: Nat Commun. 2024 Jun 4;15:4575. doi: 10.1038/s41467-024-48579-5 (PMC11150404; doi:10.1038/s41467-024-48579-5)
Supplement: Supplementary file 8 — Reporting Summary [file 41467_2024_48579_MOESM8_ESM.pdf]

Corresponding author(s): M. Gabriele Bixel, Ralf H. Adams

Last updated by author(s): Apr 8, 2024

Reporting Summary

Nature Portfolio wishes to improve the reproducibility of the work that we publish. This form provides structure for consistency and transparency in reporting. For further information on Nature Portfolio policies, see our Editorial Policies and the Editorial Policy Checklist.

Statistics

For all statistical analyses, confirm that the following items are present in the figure legend, table legend, main text, or Methods section.

n/a	Confirmed	
☐	☒	The exact sample size (*n*) for each experimental group/condition, given as a discrete number and unit of measurement
☐	☒	A statement on whether measurements were taken from distinct samples or whether the same sample was measured repeatedly
☐	☒	The statistical test(s) used AND whether they are one- or two-sided<br>*Only common tests should be described solely by name; describe more complex techniques in the Methods section.*
☐	☒	A description of all covariates tested
☐	☒	A description of any assumptions or corrections, such as tests of normality and adjustment for multiple comparisons
☐	☒	A full description of the statistical parameters including central tendency (e.g. means) or other basic estimates (e.g. regression coefficient) AND variation (e.g. standard deviation) or associated estimates of uncertainty (e.g. confidence intervals)
☐	☒	For null hypothesis testing, the test statistic (e.g. *F*, *t*, *r*) with confidence intervals, effect sizes, degrees of freedom and *P* value noted<br>*Give P values as exact values whenever suitable.*
☒	☐	For Bayesian analysis, information on the choice of priors and Markov chain Monte Carlo settings
☒	☐	For hierarchical and complex designs, identification of the appropriate level for tests and full reporting of outcomes
☒	☐	Estimates of effect sizes (e.g. Cohen's *d*, Pearson's *r*), indicating how they were calculated

Our web collection on statistics for biologists contains articles on many of the points above.

Software and code

Policy information about availability of computer code

Data collection	Multiphoton immunifluorescence images were aquired with TriM Scope II (LaVision BioTec) and its microcope lmspector acquisition software.<br>RNA sequencing was done using NestSeqS00, MiSeq (lllumina).
Data analysis	Multiphoton images were analyzed with ImageJ (open source NIH software, http://imagej.nih.gov/ij). Data analysis and statistical tests were done with ImageJ, AngioTool (Version 0.6a, open source NIH software, http://angiotool.nci.nih.gov) and GraphPad Prism (Version 7).<br>Single cell RNA sequencing data analysis:<br>Sequencing data of FASTQ format were processed with BD Rhapsody WfA Analysis pipeline (version 1.0) on Seven Bridges Genomics on line platform (SevenBridges) and expression matrix were used for further data analysis. Data normalization, dimensionality reduction and visualization were performed using Seurat (version 4.3.0).<br>FindlntegrationAnchors and IntegrateData with default options were used for the data integration. Statistically significant principal components were determined by Jackstraw method and the first 7 principal components were used for UMAP non-linear dimensional reduction.<br>Clustering & Visualisation:<br>Unsupervised hierarchical clustering analysis was performed using FindClusters function in Seurat package. We tested different resolutions between 0.1 ~ 0.9 and selected the final resolution using clustree R package to decide the most stable as well as the most relevant for our previous knowledges. Cellular identity of each cluster was determined by finding cluster-specific marker genes using FindAllMarkers function. with minimum fraction of cells expressing the gene over 25% (min.pct=0.25), comparing those markers to known cell type-specific genes from previous studies and further confirmed using the R package SingleR, which compares the transcriptome of each single cell to reference datasets to determine cellular identity.

For manuscripts utilizing custom algorithms or software that are central to the research but not yet described in published literature, software must be made available to editors and reviewers. We strongly encourage code deposition in a community repository (e.g. GitHub). See the Nature Portfolio guidelines for submitting code & software for further information.

Data

Policy information about availability of data

All manuscripts must include a data availability statement. This statement should provide the following information, where applicable:

- Accession codes, unique identifiers, or web links for publicly available datasets
- A description of any restrictions on data availability
- For clinical datasets or third party data, please ensure that the statement adheres to our policy

We have deposited the scRNAseq data in a public repository with the following accession codes GSE154247 and GSE262350
A public Cell Viewer to analyze the scRNAsec data has been provided under: https://single-cell.mpi-muenster.mpg.de/o/calvarial-lesion-2023

Research involving human participants, their data, or biological material

Policy information about studies with human participants or human data. See also policy information about sex, gender (identity/presentation), and sexual orientation and race, ethnicity and racism.

Reporting on sex and gender	Study does not involve human participants, their data or human biological material
Reporting on race, ethnicity, or other socially relevant groupings	N/A
Population characteristics	N/A
Recruitment	N/A
Ethics oversight	N/A

Note that full information on the approval of the study protocol must also be provided in the manuscript.

Field-specific reporting

Please select the one below that is the best fit for your research. If you are not sure, read the appropriate sections before making your selection.

☒ Life sciences ☐ Behavioural & social sciences ☐ Ecological, evolutionary & environmental sciences

For a reference copy of the document with all sections, see nature.com/documents/nr-reporting-summary-flat.pdf

Life sciences study design

All studies must disclose on these points even when the disclosure is negative.

Sample size	Sample sizes were determined based on sample sizes from previous similar experiments (Bixel et al. Cell Reports 2017)
Data exclusions	No data was excluded
Replication	scRNA-seq experiments were performed in duplicates. We performed at least three biologically independent experiments to ensure reproducibility. Experimental observations were reproducible and successful with comparable results.
Randomization	No formal method of randomization was needed. All experiments involving wildtype and mutant mice were performed on inbred C57B16 strain with female mice of same age group. For injury models age matched female mice were used.
Blinding	Blinding was not possible because the genetic phenotype was obvious from the image data.

Reporting for specific materials, systems and methods

We require information from authors about some types of materials, experimental systems and methods used in many studies. Here, indicate whether each material, system or method listed is relevant to your study. If you are not sure if a list item applies to your research, read the appropriate section before selecting a response.

Materials & experimental systems

n/a	Involved in the study
☐	☒ Antibodies
☒	☐ Eukaryotic cell lines
☒	☐ Palaeontology and archaeology
☐	☒ Animals and other organisms
☒	☐ Clinical data
☒	☐ Dual use research of concern
☒	☐ Plants

Methods

n/a	Involved in the study
☒	☐ ChIP-seq
☒	☐ Flow cytometry
☒	☐ MRI-based neuroimaging

Antibodies

Antibodies used

All antibody details (clone and manufacturer) are included in methods and also listed below:
Primary antibodies:
rat monoclonal anti-Endomucin (V.7C7) (Santa Cruz, Cat# sc-65495), 1:200
goat polyclonal anti-CD31 (R&D, Cat# AF3628), 1:200
goat anti-Pdgfrb (R&D, Cat# AF1042); 1:200
rabbit polyclonal anti-Osterix (Abeam, Cat#ab22552), 1:200
rabbit polyclonal anti-Acan (Milipore, Cat#AB1031), 1:200
rabbit anti-vATPaseBl/B2 (Abeam, Cat#200839); 1:200
rabbit polyclonal anti-Runx2 (Abeam, Cat#192256)), 1:100
Species-specific Alexa Fluor secondary antibodies, all from Thermo Fischer:
Alexa Fluor 594 A21209 Thermo Fischer Scientific, 1:200
Alexa Fluor 647 A31573 Thermo Fischer Scientific, 1:200
Alexa Fluor 647 A21447 Thermo Fischer Scientific, 1:200
Alexa Fluor 488 A21208 Thermo Fischer Scientific, 1:200

Validation

All antibodies used in the study have been commercially available, previously used repeatedly with similar results and published by our group (Sivaraj et al. 2022, Nat. Comm. 13, 571, Bixel et al. 2017, Cell Reports 18, 1804). The complete information and all further validation information for each antibody can be found in our publications or alternatively, on the manufacturer's website.

Animals and other research organisms

Policy information about studies involving animals; ARRIVE guidelines recommended for reporting animal research, and Sex and Gender in Research

Laboratory animals

All animals used in this study are Mus musculus species, C57/BL6 background strain independent of genotype. All mice are adult females (8-12 weeks) and were maintained in pathogen free standard condition as mentioned in the animal guidelines.
Transgenic mice were generated from our laboratory, CdhS(PAC)-CreERT2, Pdgfrb-Cre ERT2, D114 lox/lox mice; and Flkl-GFP, Sp7-mCherry were purchased from Jackson Laboratory.
Our mice were kept in individually ventilated cages (IVC), with constant access to food and water under a 14h light and 10h dark cycle regime. Air flow, temperature (22°C +/-1.5°C) and humidity (55–60%) were controlled by an air management system. Animals were checked daily and maintained in specific pathogen-free (SPF) conditions. Sufficient nesting material and environmental enrichment was provided.

Wild animals

No wild animals were used in the study.

Reporting on sex

The occurrence of fractures or the course of fracture healing is in principle independent of gender or ethnic group. However, it is known that sex hormones such as estrogen or testosterone have a strong influence on bone metabolism and regeneration. In order to minimize the number of animal experiments and possible increased variation in the results when using both sexes, we only used female animals in this study. Preliminary experiments with female and male mice showed no difference in growing blood vessels close to the growth plate in developing femurs.

Field-collected samples

No field collected samples were used.

Ethics oversight

All animal experiments were performed according to the international guidelines and laws, approved by local animal ethical committee University of Muenster and Max Planck Institute for Molecular Biomedicine with premissions granted by the Landesamt fur Natur, Umwelt und Verbraucherschutz (LAN UV) of North Rhine-Westphalia. Germany.

Note that full information on the approval of the study protocol must also be provided in the manuscript.

Plants

Seed stocks	*Report on the source of all seed stocks or other plant material used. If applicable, state the seed stock centre and catalogue number. If plant specimens were collected from the field, describe the collection location, date and sampling procedures.*
Novel plant genotypes	*Describe the methods by which all novel plant genotypes were produced. This includes those generated by transgenic approaches, gene editing, chemical/radiation-based mutagenesis and hybridization. For transgenic lines, describe the transformation method, the number of independent lines analyzed and the generation upon which experiments were performed. For gene-edited lines, describe the editor used, the endogenous sequence targeted for editing, the targeting guide RNA sequence (if applicable) and how the editor was applied.*
Authentication	*Describe any authentication procedures for each seed stock used or novel genotype generated. Describe any experiments used to assess the effect of a mutation and, where applicable, how potential secondary effects (e.g. second site T-DNA insertions, mosiacism, off-target gene editing) were examined.*
